# Associations between Corneal Nerve Structure and Function in a Veteran Population

**DOI:** 10.3390/jcm13092513

**Published:** 2024-04-25

**Authors:** Mohammad Ayoubi, Kimberly Cabrera, Elyana VT Locatelli, Elizabeth R. Felix, Anat Galor

**Affiliations:** 1 Surgical and Research Services, Miami Veterans Administration Medical Center, 1201 NW 16th Street, Miami, FL 33125, USA; mxa1947@miami.edu (M.A.); efelix@med.miami.edu (E.R.F.); 2 Bascom Palmer Eye Institute, University of Miami Miller School of Medicine, 900 NW 17th Street, Miami, FL 33136, USA

**Keywords:** dry eye disease, corneal nerves, in vivo confocal microscopy, tear production, ocular symptoms

## Abstract

**Background:** We evaluate the relationship between corneal nerve structure and function in a veteran population. **Methods:** 83 veterans (mean age: 55 ± 5 years) seen at the Miami Veterans Affairs (VA) eye clinic were included in this study. Each individual filled out questionnaires to evaluate ocular symptoms (5-Item Dry Eye Questionnaire, DEQ5; Ocular Surface Disease Index, OSDI) and ocular pain (Numerical Rating Scale, NRS; Neuropathic Pain Symptom Inventory modified for the Eye, NPSI-Eye). The individuals also underwent an ocular surface examination that captured functional nerve tests including corneal sensation, corneal staining, and the Schirmer test for tear production. Corneal sub-basal nerve analysis was conducted using in vivo confocal microscopy (IVCM) images with corneal nerve density, length, area, width, and fractal dimension captured. IVCM and functional corneal metrics from the right eye were examined using correlational and linear regression analysis. **Results:** Most corneal structural metrics were not related to functional metrics, except for weak correlations between various IVCM metrics and tear production. In addition, corneal nerve fiber area was positively related to corneal sensation (r = 0.3, *p* = 0.01). On linear regression analyses, only the corneal fractal dimension remained significantly related to tear production (β = −0.26, *p* = 0.02) and only the corneal nerve fiber area remained significantly related to corneal sensation (β = 0.3, *p* = 0.01). **Conclusions:** Most corneal nerve structural metrics did not relate to functional metrics in our veteran population, apart from a few weak correlations between structural metrics and tear production. This suggests that using corneal nerve anatomy alone may be insufficient for predicting corneal function.

## 1. Introduction

The cornea plays many critical roles including providing a protective barrier against infection and acting as an anterior refractive surface for the eye [1]. Corneal nerves are important players in allowing the cornea to perform these functions as they continuously sense the environment and elicit a response to thermal, mechanical, and chemical stimuli. Corneal nerves are also involved in the maintenance of healthy tear production and the blink reflex, and they release trophic factors that support epithelial health and allow appropriate wound healing [2]. Corneal nerve abnormalities have been detected in some subtypes of dry eye disease (DED), a heterogeneous set of conditions characterized by a loss of homeostasis of the tear film and accompanied by ocular symptoms, in which tear film instability and hyperosmolarity, ocular surface inflammation and damage, and neurosensory abnormalities play etiological roles. These abnormalities, in turn, manifest as ocular pain/discomfort and/or visual disturbances [3].

Several diagnostic tests have been used to examine corneal nerve structure and function. In vivo confocal microscopy (IVCM) is often used to examine corneal nerve structure, with most studies focusing on nerves at the level of the sub-basal epithelial layer [4,5,6]. For example, a French study assessed sub-basal corneal nerves using the Heidelberg Retina Tomograph (HRT) in 12 individuals with aqueous tear-deficient (ATD) DED (symptoms, tear film instability, staining ≥ 2, AND Schirmer ≤ 10 mm). The DED group had decreased corneal nerve density (9.43 ± 2.6 vs. 15.96 ± 2.4 mm/mm^2^, *p* < 0.0001) and a decreased number of corneal nerves (24.73 ± 8.0 vs. 39.06 ± 7.3 n/mm^2^, *p* = 0.001) compared to controls [7]. Similar patterns have been found across studies that examined nerve density in ATD [8], but a similar pattern was not consistently noted in other DED subtypes including evaporative DED. This was demonstrated in an Indian study (n = 80) that assessed sub-basal corneal nerves using IVCM (HRT) in individuals with evaporative DED (symptoms, TBUT < 10 s, AND Schirmer > 10 mm) and found no significant differences in nerve density compared to controls [9]. Overall, these findings are in concordance with a prior review conducted by our group which noted heterogeneous relationships between corneal nerve anatomy and various DED etiologies [10].

Several tests have been used as surrogate markers of corneal nerve function in DED including patient-reported pain symptoms, tear production, and corneal sensitivity, again with heterogeneity noted across and within DED etiologies. With regards to functional metrics, it has been noted that a significant proportion of individuals complain of various ocular pain symptoms, with a prevalence of ~15–50% varying across different studies and demographics globally [11]. Some, but not all, of these individuals have reduced tear production, typically measured using Schirmer strips [11]. Conversely, some individuals have decreased tear production yet no ocular pain symptoms [11]. In a similar manner, corneal sensitivity has been assessed in DED with some studies noting hyposensitivity in DED [7], while others noted hypersensitivity in symptomatic disease [12]. For example, the abovementioned French study found that individuals with ATD DED had significantly decreased corneal sensitivity (Cochet–Bonnet) compared to controls (5.00 ± 0.83 vs. 5.88 ± 0.22 cm, *p* = 0.006) [7]. Similar findings have been noted in evaporative DED. A Spanish study examined 44 individuals with TBUT ≤ 6 s and found reduced corneal sensitivity (higher thresholds on Belmonte) in DED compared to controls (152.6 ± 33.8 vs. 109.0 ± 23.3 mL/min, *p* < 0.001) [13]. However, discrepancies exist across the literature. An American study (n = 66) found that individuals with evaporative DED (symptoms, TBUT ≤ 5 s, and staining ≥ 2) had increased corneal sensitivity (Belmonte) compared to controls (34.60 ± 21.09 vs. 61.50 ± 20.07 mL/min, *p* < 0.05) [14]. Overall, similar to nerve anatomy, variability in corneal nerve function has been noted across DED populations and studies.

Some studies have also examined how corneal nerve structure relates to function in DED. In the French study, corneal nerve fiber density (r = 0.64, *p* = 0.05) and nerve count (r = 0.65, *p* = 0.04) related to corneal sensitivity (Cochet–Bonnet) [7]. However, more studies are still needed to explore connections between corneal nerve anatomy and function in distinct populations. To bridge this knowledge gap, we conducted a comprehensive analysis to explore relationships between IVCM findings and functional nerve metrics in a population of veterans. Understanding the contribution of corneal nerves to DED is an important goal as this knowledge can be translated into optimized diagnostic tests and precision-based treatment plans with the goal of improved clinical outcomes. This approach not only deepens our understanding of corneal structure and function, but also enhances our understanding of potential contributions and limitations of IVCM as applied to the field of DED.

## 2. Materials and Methods

### 2.1. Study Population

A cross sectional analysis was conducted on 83 veterans who served in the Gulf War Era (GWE) and were examined at the Miami Veterans Affairs (VA) Hospital eye clinic between October 2020 and March 2022. Individuals with normal external anatomy were included in this study (e.g., eyelids, conjunctiva, and cornea). Subjects were excluded from this study if any of the following were present: use of any ocular medications, ocular diseases that could impact ocular surface health (e.g., glaucoma, retinal surgery, pterygium, and corneal edema), systemic autoimmune diseases related to DED (Sjögrens and graft versus host disease), and any health conditions that could hinder the execution of study protocols (e.g., neurological or mental health disorders that would preclude filling out questionnaires independently). Informed consent was obtained from all individuals that participated in this study. This study was approved by the Miami Veteran Affairs (VA) Institutional Review Board (IRB). This study was also conducted in accordance with the principles of the Declaration of Helsinki and complied with the requirements of the United States Health Insurance Portability and Accountability Act.

### 2.2. Data Collection

All individuals provided data regarding demographics, co-morbidities, medications, and medical diagnoses. Their mental health status was evaluated with several questionnaires including the Patient Health Questionnaire (PHQ-9) for depression [15], the Post-Traumatic Stress Disorder Checklist—Military Version (PCL-M) [16], the Modified Fatigue Impact Scale (MFIS) for fatigue [17], and the Pittsburgh Sleep Quality Index (PSQI) for sleep quality [18].

### 2.3. Ocular Examination

Ocular symptoms: All the individuals filled out standardized questionnaires to assess ocular symptoms. Dry eye symptoms were quantified using the Ocular Surface Disease Index (OSDI, range 0–100) [19] and 5-Item Dry Eye Questionnaire (DEQ5, range 0–22) [20]. Combining these two surveys allows us to examine different aspects of symptoms including pain (OSDI: soreness, grittiness; DEQ5: dryness, discomfort), visual disturbances (OSDI: poor vision, blurriness), and other aspects of disease (OSDI: environmental triggers; DEQ5: tearing). Therefore, the overall severity score for each questionnaire is a combined measure of different symptom domains.

In addition to ocular symptoms, we also assessed ocular pain using two validated pain questionnaires. Ocular pain intensity was measured with a Numerical Rating Scale (NRS, range 0–10), a common instrument used as an endpoint in clinical trials [21]. NRS scores were collected for pain felt “right now”, “average over the last week”, and “worse over the last week”. The neuropathic features of pain were assessed using the Neuropathic Pain Symptom Inventory, modified and validated for eye pain (NPSI-Eye, total score: range 0–100; sub-score range 0–10) [22].

Examination findings: A provider masked from the clinical symptoms evaluated the examination findings. Ocular signs included, in the order assessed, the following:

1. Inflammation assessed with a qualitative measure of matrix metalloproteinase 9 (MMP-9) (InflammaDry, Quidel, San Diego, CA, USA) [23] graded as 0 = none, 1 = mild, 2 = moderate, and 3 = severe;
2. Corneal sensation assessed with a cotton-tipped swap and qualitatively graded as 0 = absent, 1 = reduced, 2 = normal, and 3 = increased;
3. Tear stability measured via tear break-up time (TBUT), 5 μL of fluorescein placed, and 3 measurements recorded and averaged. A TBUT value > 8–10 s is usually considered healthy. Lower scores indicate faster tear evaporation and potential dry eye disease;
4. Fluorescein corneal staining graded using the National Eye Institute (NEI) scale [24]; graded in 5 areas on a scale of 0 to 3 and scores summed (total range of 0–15). 0 = no staining or damage, 1 = mild staining (sparse or superficial spots), 2 = moderate staining (confluent spots), 3 = severe staining (intense, confluent staining or large areas covered). A healthy result on this test would be a score of 0 in all zones, indicating no observable damage or dye uptake;
5. Pain intensity using a 0–10 NRS assessed before and 30 s after application of 10 µL of proparacaine hydrochloride 0.5% (one drop in each eye). A score of 0 indicates no pain and 10 represents the worst possible pain;
6. Tear production using the Schirmer test at 5 min, measured in mm with anesthesia for measurement of basal tear secretion. We recognize that this test does not assess reflex tear secretion but was selected to optimize comfort. Results range from 0 to 35 mm; a score below 10 mm indicates reduced tear production;
7. Intraocular pressure was measured using the Tonopen XL^®^ applanation tonometer (Reichert, Depew, NY, USA). Healthy values range from 10 to 21 mmHg.

### 2.4. Confocal Analysis

Confocal microscopy: Laser in vivo confocal microscopy (IVCM) was performed using the Rostock Cornea Module of the Heidelberg Retina Tomograph (HRT) III (Heidelberg Engineering, Heidelberg, Germany). Qualitative image analysis was conducted by a masked reviewer. Quantitative image analysis was performed via a validated automated nerve image analysis software (ACCMetrics Corneal Nerve Fiber Analyser V.2, University of Manchester, Manchester, UK) [25]. Assessed metrics include corneal nerve fiber density (number/mm^2^), defined as the total number of main nerves per square millimeter; corneal nerve fiber length (mm/mm^2^), defined as the total length of main nerves and nerve branches per square millimeter; corneal nerve branch density (number/mm^2^), defined as the total number of main nerve branches per square millimeter; corneal nerve fiber area (mm/mm^2^), defined as the total nerve fiber area [26]; corneal nerve fiber width (mm/mm^2^), defined as the average nerve fiber width [26]; and corneal nerve fiber total branch density (number/mm^2^), defined as the total number of branch points per square millimeter. Additionally, corneal nerve fractal dimension, defined as a “novel parameter that measures the structural complexity of corneal nerves”, was assessed [27]. Corneal nerve fractal dimension is a numerical measure of an image feature’s complexity and has been used to identify structural changes in various neurological diseases and, more recently, in DED [28]. The mean of the three values (one for each analysed image) obtained for each parameter was calculated for the right eye.

### 2.5. Data Analysis

Statistical analyses were performed using SPSS 28.0 (IBM Corp., Armonk, NY, USA). Descriptive statistics were used to summarize demographic and clinical data. Pearson correlations were used to examine relationships between metrics of corneal nerve structure and function. A *p*-value of <0.05 was deemed significant for all measures. In this paper, we opted to give information on all variables being compared as opposed to correcting the *p*-value (e.g., Bonferroni) since the latter methodology has its own limitations [29]. Forward linear regression analyses were used to examine relationships between corneal structure and function, while controlling for demographics, medications, and comorbidities. Missing data points were minimal, and as such, no imputation strategies were implemented. With an n of 83, we had the power to detect medium-effect sizes for correlations between corneal structural and functional metrics using the terminology of Cohen [30].

## 3. Results

### 3.1. Study Population

Our population consisted of 83 individuals. The study demographics included a mean age of 55 years old. Of the individuals, 74 (89.2%) identified as males, 48 (57.8%) as White, and 33 (39.8%) as Hispanic. Among them, 52 (63.4%) reported no previous smoking. Ocular symptoms were measured using the DEQ5 (mean 8.82 ± 4.8), OSDI (32.7 ± 25.19), NRS (right now: 1.57 ± 2.46, average of 1 week: 1.9 ± 2.35, worst in 1 week: 2.13 ± 2.66), and NPSI-Eye (15.2 ± 18.25). Most of the individuals had intact corneal sensation (85.5%), but 6% had hyposensitivity and 8.4% had hypersensitivity in the right eye. Despite the high burden of ocular pain symptoms and mean tear stability and production along with intraocular pressure, the values were within normal limits (Table 1).

**Figure 1:**
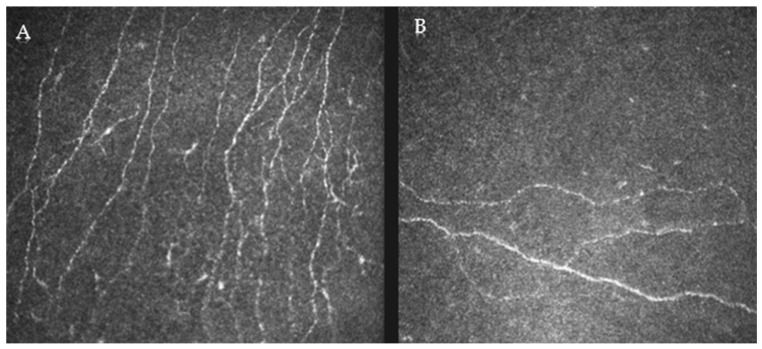
Representative images of corneal nerves on in vivo confocal microscopy (IVCM). Figure displays two representative images of corneal nerves as seen on IVCM. (**A**) shows a more robust nerve density, with a branched network of nerve fibers. (**B**) illustrates lower nerve density, where the fibers are fewer and less intricate. Both images were taken from the right eye.

The mean confocal metrics are presented in Table 2 and were calculated by averaging three distinct images from the right eye. The average corneal nerve fiber density was 13.45 ± 8.29 n/mm^2^, with some patients demonstrating reduced corneal nerve density (Figure 1). It should be noted that there is a lack of standard reference values for confocal parameters in the literature due to discrepancies according to the type of imaging equipment, protocols for image capture, and definitions of corneal nerve structures.

### 3.2. Corneal Structure and Function Correlations

Overall, most corneal structural metrics were not related to functional metrics, with the exception of weak correlations between various IVCM and tear production metrics (Table 3). In addition, corneal nerve fiber area was positively related to corneal sensation (r = 0.3, *p* = 0.01).

### 3.3. Multivariable Models

Forward linear regression analyses were performed while controlling for demographics (age, gender, and race), smoking status (previous, current, and never), medication use (antidepressants, antianxiety, and antihistamines), mental health (depression and PTSD), and co-morbidities (hypertension, hyperlipidemia, diabetes mellitus (DM), and sleep apnea). Of all the IVCM metrics, only corneal fractal dimension remained significantly related to tear production (β = −0.26, *p* = 0.02), and only corneal nerve fiber area remained significantly related to corneal sensation (β = 0.3, *p* = 0.01) (Table 4). No other variables remained significantly related to tear production or corneal sensation.

## 4. Discussion

To conclude, our study comprehensively examined the relationships between metrics of corneal nerve structure and function in a unique population of individuals without autoimmune associated DED. We found that most corneal structural metrics did not correlate with functional metrics, with the exception that tear production was negatively associated with corneal nerve fiber length, density, and fractal dimension, and positively associated with width (−0.3 < r < 0.3 for all). In addition, corneal nerve fiber area was positively but weak to moderately associated with corneal sensation (r = 0.30). No other structural metrics were related to corneal sensation.

Our findings share similarities and differences with prior studies that examined relationships between IVCM metrics and corneal function. First, this question has been examined in healthy populations. In a New Zealand study of 60 individuals without ocular disease, weak correlations were noted between corneal structural metrics (measured with Confoscan microscopy) and function (measured with a non-contact corneal aesthesiometer). Specifically, a weak correlation was noted between corneal nerve density and temporal corneal sensitivity (r = −0.18, *p* = 0.05), indicating lower temporal sensitivity in individuals with lower nerve density. However, significant relationships were not noted in other areas of the cornea (central, inferior, nasal, and superior: *p* > 0.05 for all) [31]. Second, relationships between IVCM metrics and corneal function have been examined in various disease states, including DM. A Finnish study assessed 23 individuals with type 1 DM but no ocular disease and found moderate correlations between corneal structure (measured with a Tandem Scanning microscope) and function (measured with Cochet–Bonnet). Specifically, corneal nerve density was moderately positively correlated with corneal sensitivity (r = 0.4, *p* = 0.048), indicating lower corneal sensitivity in those with lower nerve density [32]. These studies reveal varying relationship strengths across different health contexts [10].

This question has also been examined in individuals with DED, both with ATD and evaporative DED. A Chinese study examined this question in 57 individuals with non-Sjögren DED (symptoms AND Schirmer < 5 mm and/or TBUT < 10 s). Similar to our study, no significant relationships were noted between most IVCM metrics (corneal nerve density, number, and width) and functional metrics (corneal sensitivity measured with Cochet–Bonnet, TBUT, and tear production). In contrast to our study, a moderate correlation was noted between nerve density and central corneal sensitivity (r = 0.38, *p* = 0.02), indicating lower central sensitivity in those with lower nerve density. However, when the group reanalysed the data using the mean corneal sensitivity across five corneal regions (central, superior, inferior, nasal, and temporal), the relationship vanished. In addition, unlike our study, corneal nerve fiber length was moderately correlated with ocular symptoms (OSDI: r = −0.39, *p* = 0.01), indicating more symptoms in individuals with shorter nerves [33].

Studies have also examined this question in specific DED populations, such as Sjögrens DED (SS-DED) and DM. A French study of 30 individuals with SS-DED assessed corneal structure (measured with HRT) and found a moderate relationship between corneal nerve density (r = 0.54, *p* = 0.002) and number (r = 0.61, *p* < 0.001) and corneal sensitivity (measured with Cochet–Bonnet), indicating lower sensitivity in individuals with lower nerve density and number [34]. However, these findings have not been uniform across studies. In a different French study of 71 individuals with SS-DED, no correlations were noted between structural metrics (measured with HRT) and functional metrics (Cochet–Bonnet, TBUT, fluorescein staining, tear production). The exception was a weak correlation between corneal nerve density and TBUT (r = 0.31, *p* = 0.008), indicating decreased tear stability in individuals with lower nerve density [35]. With respect to DM and DED, a Chinese study of 62 individuals with type 1 DM and DED (≥2 of: OSDI > 13, TBUT < 10 s, or corneal staining > 5) noted weak to moderate correlations between corneal nerve density (measured with HRT) and both corneal sensitivity (Cochet–Bonnet: r = 0.52, *p* < 0.01) and OSDI scores (r = 0.2, *p* < 0.05). Similar relationships were noted between corneal nerve fiber length and both corneal sensitivity (r = 0.42, *p* < 0.01) and OSDI scores (r = 0.31, *p* < 0.01), indicating lower sensitivity but less symptoms in individuals with lower nerve density and length [36].

Taken together, the noted differences may be explained by different populations, different devices to measure corneal nerve anatomy (Confoscan vs. HRT) and sensitivity (non-contact vs. contact methodologies), and different methodologies to quantify nerve metrics. As suggested by a recent review, future studies are needed to standardize the terminology and analytic methods in corneal nerve imaging, with the hope that this will enhance its clinical relevance in diagnosing DED and monitoring treatment [37]. However, when considering all data concomitantly, the population with the strongest correlation between structure and function appears to be individuals with diabetes, highlighting the neurotrophic phenotype often noted in this population.

Interestingly, our findings with respect to the eye share similarities with those noted in other body areas, such as the skin. Overall, poor correlations have been noted between nerve structure and function in the skin. One comprehensive review that included a total of 111 studies of individuals with distal symmetric polyneuropathies reported heterogeneous associations between intraepidermal nerve fiber density (IENFD: measured with skin biopsy) and various nerve function metrics, including detection and pain thresholds for cold and warmth, vibration thresholds, and mechanical thresholds. For example, only 48% of studies found significant associations between IENFD and cold detection threshold. IENFD and warmth detection threshold were more closely related, with nearly 70% of studies reporting associations between lower nerve fiber density and increased warmth detection thresholds (i.e., implying lower sensitivity). Regarding pain perception, about half of the studies showed significant associations between IENFD and heat pain thresholds, while only 38% of studies showed significant associations between IENFD and cold pain thresholds. Vibration detection thresholds were less frequently associated with IENFD, with relationships noted in 33% of studies. Closest to our testing of sensitivity, mechanical detection and pressure pain thresholds showed the least consistency across studies, suggesting weak relationships between these entities and perhaps explaining the weak correlation noted between IVCM and mechanical thresholds in our study. While this review noted the general direction of the relationships observed, no comments were made regarding the strength of association [38]. Future studies are needed to examine whether hot and cold corneal thresholds would be more robustly associated with corneal nerve anatomy, as has been found in the skin.

As with all studies, our findings must be considered in light of its limitations. First, our population consisted of South Floridian veterans, of which a majority were male. As such, our outcomes may not be applicable to other populations. Second, our cross-sectional analysis precludes an examination of causality or directionality in the few relationships observed. Third, although minimal, missing data may have introduced potential biases in our findings. Fourth, we elected to measure corneal sensitivity in a qualitative manner which is less precise than prior studies that used the Cochet–Bonnet and Belmonte aesthesiometers. Fifth, participants utilizing prescribed ocular medications were excluded from our study, thus limiting the generalizability of our findings to other DED populations. Despite these limitations, this study offers caution in using corneal nerve anatomy, as it is currently captured and reported (e.g., density and branching), to predict function. While more robust findings were noted in specific conditions such as DM, weak relationships were noted in healthy individuals and non-SS DED. While IVCM is invaluable for detailed visualization of corneal structure, its current limitation in assessing functional outcomes highlights a gap in our diagnostic arsenal. As such, there is a need to deepen our understanding of IVCM utility, including assessing novel parameters that may more closely relate to function (e.g., microneuromas [39]), while at the same time developing novel tests that directly assess function, with the hope that this information can be translated into better therapeutic algorithms that improve the quality of life for our patients.

## Figures and Tables

**Table 1:** Demographics, co-morbidities, medications, ocular symptoms, and ocular signs in the study population.

Demographics	Population (n = 83)
**Age, years, mean ± SD, range**	55 ± 5, 48–67
**Sex, male, % (n)**	89.2% (74)
**Race, % (n)**	
White	57.8% (48)
Black	38.6% (32)
Other	3.60% (3)
**Ethnicity, % (n)**	
Hispanic	39.8% (33)
Non-Hispanic	60.2% (50)
**Non-ocular comorbidities, % (n)**	
Hypertension	38.6% (32)
Hyperlipidemia	50.6% (42)
Diabetes mellitus	16.9% (14)
Sleep apnea	59.8% (49)
Benign prostatic hyperplasia	13.6% (11)
**Smoking status, % (n)**	
Never	63.4% (52)
Former	19.5% (16)
Current	17.1% (14)
**Mental health, mean ± SD**	
Depression, PHQ-9 (range 0–27)	10.16 ± 7.45
Fatigue, MFIS (range 0–84)	37.46 ± 23.12
PTSD, PCL-M (range 17–85)	44.74 ± 17.78
Sleep, PSQI (range 0–21)	11.94 ± 4.57
**Oral medications, % (n)**	
Antianxiety	14.8% (12)
Antidepressant	27.2% (22)
Antihistamine	28.4% (23)
Betablocker	13.6% (11)
Fish oil	24.7% (20)
Multivitamin	32.9% (27)
NSAID	37.0% (30)
Statins	43.2% (35)
**Ocular symptoms, mean ± SD**	
DEQ5	8.82 ± 4.80
OSDI	32.7 ± 25.19
NPSI-Eye	15.2 ± 18.25
NRS (right now)	1.57 ± 2.46
NRS (average of 1 week)	1.90 ± 2.35
NRS (worst in 1 week)	2.13 ± 2.66
Ocular surface exam *	
Inflammation, MMP9, mean ± SD	0.84 ± 0.91
Corneal sensation, % (n)	
Decreased	6% (5)
Normal	85.5% (71)
Increased	8.40% (7)
Tear break up time, seconds, mean ± SD	9.57 ± 4.26
Corneal staining, mean ± SD	0.73 ± 1.42
Schirmer score, mean ± SD	16.6 ± 9.48
Intraocular pressure, mmHg, mean ± SD	17.24 ± 3.22

SD = standard deviation; n = number in each group; PHQ-9 = Patient Health Questionnaire; MFIS = Modified Fatigue Impact Scale; PTSD = post-traumatic stress disorder; PCL-M = PTSD CheckList—Military Version; PSQI = Pittsburgh Sleep Quality Index; NSAID = Non-Steroidal Anti-Inflammatory; DEQ5 = 5-Item Dry Eye Questionnaire; OSDI = Ocular Surface Disease Index; NPSI-Eye = Neuropathic Pain Symptom Inventory—Eye; NRS = Numeric Rating Scale for eye pain; MMP9 = Matrix-Metalloproteinase 9. * All values from the right eye.

**Table 2:** Mean corneal structural metric values in the study population.

Corneal Structure Metrics *	Mean ± SD
Corneal nerve fiber density, number/mm^2^	13.45 ± 8.29
Corneal nerve fiber length, mm/mm^2^	9.79 ± 3.96
Corneal nerve branch density, number/mm^2^	13.03 ± 11.90
Corneal nerve fiber area, mm/mm^2^	0.01 ± 0.01
Corneal nerve fiber width, mm/mm^2^	0.02 ± 0.00
Corneal nerve fiber total branch density, number/mm^2^	25.68 ± 19.07
Corneal fractal dimension	1.43 ± 0.05

SD = standard deviation. * All values from the right eye, average of 3 distinct images.

**Table 3:** Correlations between corneal structural and functional metrics.

	CNFD	CNBD	CNFL	CTBD	CNFA	CNFW	CFD
**Ocular Symptoms**							
Dry Eye Questionnaire 5	−0.11	−0.14	−0.04	−0.03	0.15	0.04	−0.06
Ocular Surface Disease Index	0.05	0.03	0.09	0.02	0.01	−0.14	0.05
NRS Now	0.06	0.08	0.12	0.13	0.08	0.03	0.05
NRS Avg. Week	0.07	0.02	0.12	0.10	0.08	0.00	0.07
NRS Worst Week	0.03	0.05	0.12	0.10	0.08	−0.02	0.09
NPSI-Eye	−0.02	0.05	0.08	0.14	0.15	−0.06	0.02
Ocular Surface Exam *							
Inflammation	−0.08	−0.15	−0.11	−0.20	0.01	−0.09	−0.15
Corneal Sensation	−0.05	0.00	−0.01	0.13	0.30 **	−0.09	−0.04
Tear Break Up Time	−0.03	0.15	0.04	0.03	−0.17	−0.10	−0.05
Corneal Staining	−0.09	−0.11	−0.05	−0.12	−0.07	−0.03	−0.07
Tear Production	−0.25 **	−0.16	−0.26 **	−0.12	0.01	0.23 **	−0.26 **
Intraocular Pressure	−0.14	−0.11	−0.17	−0.10	0.22	0.06	−0.20

CNFD = Corneal nerve fiber density, number/mm^2^; CNBD = Corneal nerve branch density, number/mm^2^; CNFL = Corneal nerve fiber length, mm/mm^2^; CTBD = Corneal nerve fiber total branch density number/mm^2^; CNFA = Corneal nerve fiber area, mm/mm^2^; CNFW = Corneal nerve fiber width, mm/mm^2^; CFD = Corneal fractal dimension; NRS = Numeric Rating Scale for eye pain; NPSI-Eye = Neuropathic Pain Symptom Inventory—Eye. * All values from the right eye. ** Correlation is significant at the 0.05 level (2-tailed).

**Table 4:** Linear regression analysis between corneal structural and functional metrics.

		Beta	*p* Value
Tear Production	Corneal Fractal Dimension	−0.26	0.02
Corneal Sensation	Corneal Nerve Fiber Area	0.30	0.01

## Data Availability

The data presented in this study may be available on request from the corresponding author.
